# Subfunctionalization of Duplicated Zebrafish *pax6* Genes by *cis*-Regulatory Divergence

**DOI:** 10.1371/journal.pgen.0040029

**Published:** 2008-02-15

**Authors:** Dirk A Kleinjan, Ruth M Bancewicz, Philippe Gautier, Ralf Dahm, Helia B Schonthaler, Giuseppe Damante, Anne Seawright, Ann M Hever, Patricia L Yeyati, Veronica van Heyningen, Pedro Coutinho

**Affiliations:** 1 Medical Research Council (MRC) Human Genetics Unit, Western General Hospital, Edinburgh, United Kingdom; 2 Department of Genetics, Max-Planck Institute for Developmental Biology, Tübingen, Germany; 3 Department of Science and Biomedical Technology, University of Udine, Udine, Italy; The Jackson Laboratory, United States of America

## Abstract

Gene duplication is a major driver of evolutionary divergence. In most vertebrates a single *PAX6* gene encodes a transcription factor required for eye, brain, olfactory system, and pancreas development. In zebrafish, following a postulated whole-genome duplication event in an ancestral teleost, duplicates *pax6a* and *pax6b* jointly fulfill these roles. Mapping of the homozygously viable eye mutant *sunrise* identified a homeodomain missense change in *pax6b*, leading to loss of target binding. The mild phenotype emphasizes role-sharing between the co-orthologues. Meticulous mapping of isolated BACs identified perturbed synteny relationships around the duplicates. This highlights the functional conservation of *pax6* downstream (3′) control sequences, which in most vertebrates reside within the introns of a ubiquitously expressed neighbour gene, ELP4, whose *pax6a*-linked exons have been lost in zebrafish. Reporter transgenic studies in both mouse and zebrafish, combined with analysis of vertebrate sequence conservation, reveal loss and retention of specific *cis*-regulatory elements, correlating strongly with the diverged expression of co-orthologues, and providing clear evidence for evolution by subfunctionalization.

## Author Summary

Studying the zebrafish small eyed mutant “sunrise,” we identified the causative amino acid change in the *pax6b* gene. This mutation leads to reduced DNA binding capacity. There are two closely related pax6 genes in zebrafish, *pax6a* and *pax6b*, which arose following a whole-genome duplication event about 350 million years ago; they map to different chromosomes. Each copy is now associated with a different subset of the neighbouring genes found associated with all vertebrate single-copy *Pax6* genes. The expression patterns of *pax6a* and *pax6b* have diverged from each other since the duplication event. Some division of labour has emerged: *pax6b* is less widely expressed in the brain than *pax6a*, but only *pax6b* is found in the developing pancreas. Multiple evolutionarily conserved regulatory elements (enhancers) control these expression patterns, which can be recapitulated in transgenic animals. Some enhancer elements lie more than 150 kb outside the transcribed gene region, inside the introns of unrelated neighbouring genes. Such juxtaposition imposes the need to conserve gene order in many vertebrate species. Genome duplication releases the constraint for retaining all neighbouring genes. Thus, *pax6a* has lost the coding region of its immediate neighbours, although it retains most of the brain-specific regulatory domains. Duplication also allows some orderly changes in the exact role of each regulatory component, as long as the two duplicates can, together, ensure the complex expression pattern required for complete function. We demonstrate functional loss of a brain element downstream of *pax6b*, while an upstream pancreas enhancer element has evolved in a more complex way.

## Introduction

Complex spatiotemporal control is required to regulate the expression of major tissue-specific transcription factors and signaling molecules which fulfill multiple roles in developmental patterning [1,2]. Accumulating evidence suggests that evolutionarily conserved non-coding elements orchestrate this process [3–5]. In addition to transgenic reporter studies in multiple species [6–12], human disease-associated genomic rearrangements (reviewed in refs [2,13]) and mutations [8,14], as well as natural and targeted deletions in mice [15–17], have contributed to our current understanding of how these regulatory elements control gene expression. Alteration of *cis*-regulatory function has been proposed as an important mechanism for evolutionary divergence [18]. Gene duplication provides an opportunity for such diversification without major penalties for loss of function [19]. About 350 million years ago, whole genome duplication occurred in an ancestor of teleost fishes, followed by variable loss of duplicated segments, and this process has been suggested to account for extensive speciation [20–23], so that half of all vertebrate species are teleost fish. Mechanisms for retention of duplicated copies remain controversial. Gain of new functions (neofunctionalization) has been widely proposed, but recently subfunctionalization, a more neutral partitioning of ancestral functions between duplicates, has been suggested [20,24]. A number of key transcription factors represented only once in mammalian genomes, are present as duplicates in zebrafish (Danio rerio). Often different loci are duplicated in other teleosts, such as medaka (Oryzias latipes) and pufferfish (Fugu rubripes). Where duplicate (or triplicate) genes have been examined, they reveal functional divergence, although the mechanisms have generally not been explored [25].

Our interest in developmental eye anomalies in humans and model systems, led us to map the homozygous viable and fertile zebrafish microphthalmia mutation *sunrise* (*sri*) [26]. The original mutant was isolated in the Tubingen ENU mutagenesis screen [27]. We identified a deleterious missense mutation in the homeodomain of the *pax6b* gene. To assess gene function in the context of observed phenotype, this finding triggered a comparison of expression patterns and regulatory control elements for the duplicated *pax6a* and *pax6b* genes, with each other and with other vertebrates where generally only a single orthologue is found.

PAX6 is a highly conserved protein, with paired- and homeodomain DNA-binding regions; it functions as a transcription factor with a major role in eye and brain development from Drosophila to humans [28,29]. Homozygous loss-of-function mutations lead to absence of the eye and lethal brain anomalies in humans, mice and flies [30–33]. Heterozygous null mutations (haploinsufficiency) are associated with human aniridia and murine microphthalmia (Small eye); missense changes lead to related, sometimes more severe, eye defects [29]. Associated brain anomalies have also been described in both human and mouse heterozygotes [34,35]. Its expression pattern suggested, and whole animal functional studies have shown, that Pax6 also plays an essential role in the development and adult maintenance of pancreatic endocrine cells [36]. When the conditionally targeted *Pax6* gene is homozygously inactivated in the mouse pancreas at E9.5, the pups die of severe diabetes between postnatal days 3 and 6.

The complex spatiotemporal expression of Pax6 is controlled by an extensive downstream regulatory region [6] whose existence was heralded by haploinsufficient aniridia-associated chromosomal disruptions positioned more than 150 kb downstream of *PAX6* [37]. DNaseI hypersensitivity analysis and strong, consistently patterned enhancer function in reporter transgenic animals confirmed and elaborated the predicted regulatory element organization [6,7,38]. In many cases regulatory element function can be predicted by non-coding region genomic sequence conservation in mammals or, more broadly, in vertebrates [7,10,12,38–40].

It was against this background that we examined the *sri* mutant and the regulation of the two *pax6* co-orthologues, to gain insight into the evolutionary conservation and divergence of these duplicate genes.

## Results/Discussion

### Mapping and Identification of *pax6b* as the sri Gene; Functional Analysis of Wild Type and Mutant Proteins and Definition of the Phenotype

**Figure 1:**
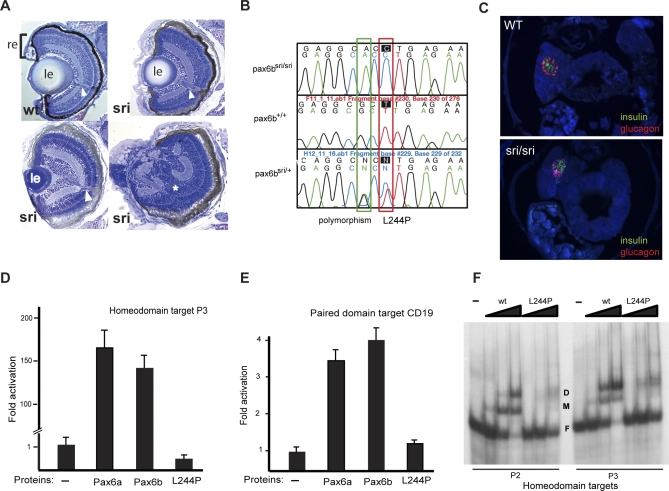
Characterisation of the *sunrise (sri)* Mutant (A) Sections through wild type and homozygous *sri* larval eyes at 5 dpf, showing variable severity from mildly reduced lens size to almost complete absence of lens and retinal malformation (∗). le, lens; re, neural retina and retinal pigment epithelium (black layer); arrowhead, optic nerve. (B) Sequence traces from *pax6b* homeodomain revealing the T to C mutation resulting in the L244P mutation. The G to A third position change two bases earlier is a polymorphism between the WIK and Tü strains. (C) Expression of known pax6 targets, glucagon and insulin, in pancreas of wild type (wt) 5 dpf fish. Expression is maintained in *sri* despite the loss-of-function homeodomain missense mutation in Pax6b, the sole *pax6* gene expressed in the pancreas. (D–F) Functional analysis of the Pax6b^sri^ (L244P) protein in comparison with wild type Pax6b and Pax6a, using luciferase reporter assays in HeLa cells and EMSA. (D) Pax6a and Pax6b proteins drive luciferase expression at comparable levels under the control of the P3 homeodomain promoter; the L244P mutant of Pax6b protein fails to activate P3. (E) Pax6a and Pax6b proteins drive comparable luciferase expression levels through the CD19 paired domain target promoter; the L244P mutant of Pax6b protein has significantly reduced activity. (F) Gel-retardation assay to analyse the binding capacity to homeodomain target binding sites P2 and P3 demonstrates reduced affinity of the L244P mutant protein compared to wild type (wt) Pax6b. D, dimeric protein-bound; M, monomeric protein-bound; and F, free labeled target oligonucleotide. Protein concentrations used 0.5, 1.5, and 4.5 μM (lanes from left to right) with 5 nM oligonucleotides.

Detailed analysis of eyes in *sri* mutant zebrafish [27], revealed a variable, but fully penetrant recessive phenotype, with abnormal lens and corneal structure leading to reduced eye size (Figure 1A). Affected homozygotes are viable and fertile, and this allowed outcrossing to the wild-type line WIK for mapping [41,42]. The uniformly heterozygous F1 siblings from this cross were interbred and the recessive phenotype was recovered, permitting the collection of phenotypically classified individuals for mapping. Standard techniques [41,42] were used to map the *sri* locus to a region, between 29.7 and 33.2 cM from the top of linkage group 7 (LG7) (Figure S1A). *pax6b* [43] was identified as a strong candidate gene. Sequencing of *sri* homozygotes and heterozygotes identified a leucine to proline missense mutation in a highly conserved residue of the *pax6b* homeodomain (Figures 1B, S1B, and S1C). The L244P alteration, in the first helix of the homeobox, is predicted to affect protein structure and function significantly [44]. A luciferase reporter assay was used to assess the regulatory capabilities of the full length Pax6a and Pax6b wild type proteins, and that of the L244P Pax6b mutant. Activation by the two wild type co-orthologues through the P3 homeodomain target [45] (Figure 1D), or the CD19 paired domain target [46] (Figure 1E), was not significantly different, in contrast to the original observations by Nornes et al [43]. However, the mutation abolished activation through the P3 homeodomain target and severely compromised CD19 paired domain function (Figure 1D and 1E) [47,48]. Binding of mutant protein to both high-affinity homeodomain target sequences P2 and P3, with different spacing between the twin sites [45], is also reduced in an electrophoretic mobility shift assay (Figure 1F). Following the identification of the mutation, the frequencies of the three expected genotypes were assessed after crossing obligate heterozygotes and found not to deviate from the expected 1:2:1 Mendelian ratios at 5 dpf, confirming the full fitness of the homozygotes at this stage (Figures 1C and S2). Survival into adulthood is good; *sri* fish are maintained as homozygotes. The viability, fertility and relatively mild eye phenotype of the homozygous *pax6b* mutant fish, led us to investigate further the role of this gene, and its co-orthologue *pax6a,* which maps to LG25 (http://zfin.org/cgi-bin/mapper_select.cgi) and Supporting Information).

### Comparison of the *pax6a* and *pax6b* Expression Patterns and Analysis of the sri Mutant

**Figure 2:**
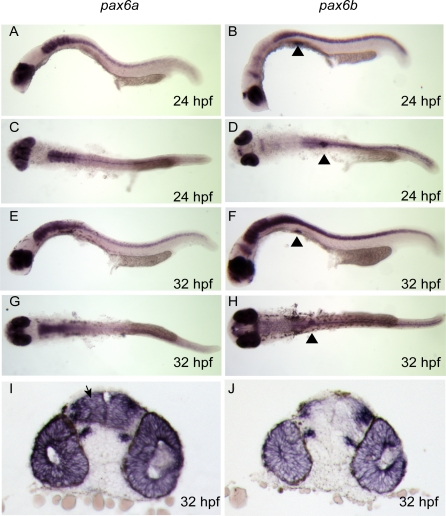
*pax6a* and *pax6b* Expression Analysis by Wholemount RNA *in situ* Hybridisation during Early Zebrafish Development *pax6a* expression is shown in (A) and (C) at 24 hpf; (A) lateral view showing expression in eye, telencephalon, diencephalon, hind brain, and neural tube (C) dorsal view; (E) and (G) lateral and dorsal view at 32 hpf, showing continuing pattern of expression, (I) Vibratome coronal section of wholemount stained embryo expressing *pax6a* in telencephalon (arrowed), diencephalon, retina and lens. (B) *pax6b* expression is seen in lateral view showing eye and optic tectum at 24 hpf, pancreas expression is highlighted using black triangles; (D) dorsal view at 24 hpf. (F) and (H) lateral and dorsal. Increasing hindbrain and neural tube expression of *pax6b* is revealed by 32 hpf embryos. (J) predominantly eye expression of *pa6b* is seen in coronal section at 32 hpf.

Overlapping divergent expression patterns have been reported for *pax6a* and *pax6b* (previously named *pax6.1* and *pax6.2* respectively) [43,49]. *pax6b* is the “minor” co-orthologue, expressed in the developing eye (retina and early lens placode), and also in thin strips of dorsal diencephalon, at the midbrain-hindbrain boundary (MHB), and in the pancreas. *pax6a* is expressed in the lens and retina, as well as more widely in the developing telencephalon, diencephalon, hind brain, and spinal cord, although not in developing pancreas [49]. The expression pattern of the co-orthologues was confirmed by RNA in situ analysis at 24 and 32 hpf (Figure 2A–2J). There is significant overlap between the territories of the co-orthologues, clearly illustrated in the sections showing *pax6a* and *pax6b* expression (Figure 2I and 2J).

**Figure 3:**
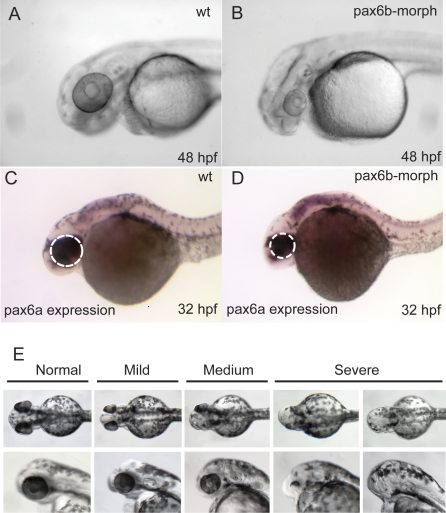
Analysis of *pax6b* Function in Zebrafish, with the Aim of Validating the Effect of the *pax6b* L244P Missense Mutation in *sri* Fish (A–D) Phenotype elicited using *pax6b* morpholinos: (A) Uninjected wild type fish at 48 hpf; fish injected with the same amount of a control scrambled morpholino also produced no effect. (B) Substantially reduced eye size and generally delayed development are observed by 48 hpf in response to injection of either of the two *pax6b* morpholinos. (C and D) At 32 hpf reduction in eye size is observed (white dotted outlines) in otherwise normal looking embryos. (E) The effects of injecting capped mRNA at levels that generally rescue the phenotype illustrating the spectrum of phenotypes to bilateral anophthalmia, observed when *pax6b* capped mRNA is injected at levels generally used for phenotype rescue experiments in zebrafish.

We set out to verify that the L244P mutation is causative for the *sunrise* phenotype. First, *pax6b* morpholino injections, using two different morpholinos, resulted in reduced eye size, a phenotype overlapping the *sri* phenotype, but somewhat more severe, as total eye size reduction (Figure 3A and 3B) rather than just small lens size [27] (Figure 1A) was observed. Morpholino injection was found to reduce protein expression proportionately to dosage (Western blot data not shown), ultimately leading to a null phenotype, while the homeodomain missense mutation may possess some residual function, even if DNA-binding capacity was strongly diminished by the mutation. This differential effect of missense and null mutations is also illustrated by the distinct phenotypes observed when patients with heterozygous missense mutations in the paired domain are compared with haploinsufficient cases ([29,50,51] and van Heyningen et al unpublished). In addition to the reduced eye phenotype, developmental delay is observed if the fish are followed for 48 hpf (Figure 3B), although expression of *pax6a* mRNA continues (Figure 3C and 3D). Simultaneous injection of *pax6a* and *pax6b* morpholinos disrupts eye development leading to microphthalmia and general developmental delay (data not shown).

Rescue of the *sri* phenotype was attempted, using capped mRNA injection into 1–2 cell stage embryos [52,53], but this led to severe developmental delay at a later stage, with reduced eye size, at mRNA levels normally used for such studies (Figure 3E). We postulate that this is because the requirement for functional Pax6 protein is both tissue-specific and highly dosage sensitive, and neither of these aspects can be controlled during mRNA-rescue by injection. Protein carrying a single altered amino-acid is usually expressed normally, probably with some intact functional domains. It is likely to be present at regular or even at elevated levels, as protein with reduced function may not be capable of driving the negative autoregulation that has been observed for Pax6 in mice [54,55]. Titration of mRNAs in wild type one- or two-cell embryos was repeated three times and the data combined (Table 1). This revealed that abnormal phenotypes begin to be elicited even with 10 pg injections of wild type *pax6b*, and increasing the amount of mRNA increases the severity score (Table 1). However the mutant L244P Pax6b-encoding mRNA consistently produces less severe phenotypes than the wild type at equivalent levels (Figure 3E; Table 1). Thus, although we were unable to rescue the effect of the missense mutation using capped mRNA injection into 1–2 cell stage mutant embryos, we show that increased dosage of wild type pax6b leads to a deleterious phenotype, and that the over-expression phenotype is less severe with an equal amount of mRNA encoding the L244P mutant protein, thus providing additional indirect evidence that this partial loss-of-function mutation is the underlying cause of the *sri* phenotype.

**Table 1:**
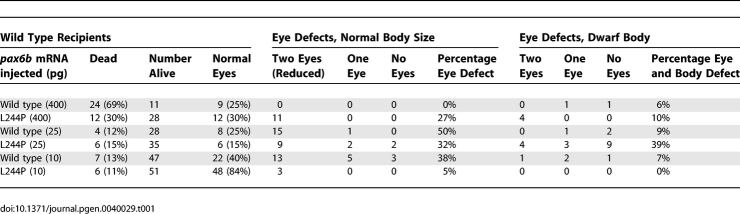
Injection of Capped mRNA into Wild Type (Tupfel Long Fin) Fish

In the mouse *Pax6* expression is observed in fetal and adult pancreas [36]. In zebrafish only *pax6b* is expressed in the developing pancreas, at least up to 48h (Figure 2), as previously reported [49] We have also explored *pax6* expression in adult zebrafish and demonstrate by RT PCR that only *pax6b* is expressed in the pancreas isolated from 6 month old wild type and *sri/sri* fish, while eyes from the same individuals express both pax6a and pax6b (Figure S3). We also showed that pax6a expression is not induced in the pancreas of the sunrise mutant. However, if only *pax6b* is expressed in the zebrafish pancreas, then the continuing normal expression of insulin and glucagon in the single-islet zebrafish pancreas of *sri* homozygotes (Figure 1C), is surprising. All known homozygous null and most missense mutations of *Pax6* in mice [56–58], as well as most reported compound heterozygotes in humans [31], and van Heyningen et al, unpublished data) die immediately postnatally, if allowed to come to term. However, there is evidence, in both mouse and humans, that rare homeodomain missense mutations in *Pax6* produce significantly milder phenotypes than nonsense mutations or paired-domain missense changes [29,51,56,59,60]. The significant but not complete abolition of homeodomain target DNA-binding, seen when the L244P mutation is assessed using EMSA (Figure 1F), is in line with this theory. Another possibility is that the paired domain, and not the homeodomain, fulfils the critical role in driving gene expression in the pancreas [61], where insulin, glucagon and somatostatin are all regulated by the −5a form of the Pax6 paired domain [62]. Although the luciferase reporter studies reveal that the *sri* homeodomain mutation severely limits paired domain binding as well (Figure 1E), this may be a cell-line-specific effect [63]. However, the converse has also been reported, namely that paired domain mutations disrupt homeodomain binding [47,48], suggesting that the Pax6 protein-DNA binding interaction requires collaboration between the paired- and homeo-domains. Interestingly, we recently identified (van Heyningen et al, unpublished) compound heterozygosity for two different *PAX6* mutations (a premature truncation and a missense change at a completely conserved arginine just N-terminal to the homeodomain) in a child who is developing normally apart from anophthalmia, suggesting that missense changes in the homeodomain region may be viable. The missense mutation carrying mother of this child has a severe eye phenotype. A similar surviving compound heterozygote case has also been reported [64].

### Evolution of Synteny Relationships around *pax6a* and *pax6b*

To gain further insight into the evolution of the duplicated zebrafish pax6a and pax6b genes, we assessed their conservation and divergence through extensive sequence comparison over coding and non-coding domains, including multiple functionally defined *cis*-regulatory elements [6,7,9,65], and through regional synteny studies. Human reticulocalbin (RCN1) was mapped between PAX6 and WT1 (Wilms tumour predisposition gene), using somatic cell hybrids [66]. Synteny conservation with mouse was noted [66], and later extended to Fugu [67]. Subsequently, the transcriptional elongation protein 4 (ELP4), a component of the elongating RNA polymerase II complex, was identified as the neighbour gene downstream of PAX6 [68]. In addition to well-defined regulatory elements upstream of promoters P0 and P1 [39, 65,69,] and within introns [9,65,70,71], a number of conserved elements required for correct spatiotemporal expression of PAX6 were found to map downstream of PAX6 within introns of ELP4, thus creating a requirement for obligate synteny conservation between the two genes [67,68]. Several of the conserved elements have been shown to function as transgenic enhancers capable of driving lacZ reporter expression, in spatiotemporal patterns corresponding to subsets of the complete Pax6 expression pattern [6,7,38].

**Figure 4:**
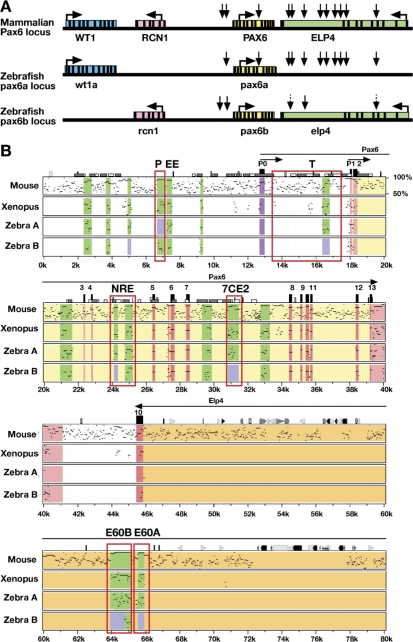
Comparative Linkage and Sequence Analysis of *pax6* Loci (A) Synteny relationships of *pax6* with its nearest neighbours. In mammals *pax6* is flanked by *rcn1* and *elp4* genes with wt1 located further upstream. Horizontal arrows show direction of transcription. Vertical arrows indicate the positions of a subset of the *pax6 cis*-regulatory elements, many located downstream of *pax6*, within introns of the adjacent *elp4* gene. Dotted arrows denote partially conserved elements. Zebrafish LG25 retains *pax6a* synteny with *wt1a* and with the majority of *pax6 cis*-regulatory elements, but *rcn1* and *elp4* coding exons have been lost. On LG7 the *pax6b* locus and *rcn1* and *elp4* exons have been retained, but many of the *pax6b* control elements, as well as the wt1 homolog, have been lost. (B) PIP plot comparing the *PAX6* locus in human with mouse, *Xenopus* and zebrafish *pax6a* and *pax6b*. Exons are shown in red, untranslated regions in pink, conserved *cis*-regulatory elements are shown in green, while absence of significant sequence conservation is indicated by a light blue box, and promoter P0 is demarcated in purple. The *cis*-regulatory elements discussed in the text are surrounded by open red rectangles. Enhancers: P, pancreas; T, telencephalon; NRE, neuroretina; 7CE2, diencephalon; E60B, ultraconserved enhancer with multiple brain specific functions (D. McBride, D. A. Kleinjan, and V. van Heyningen unpublished data); E60A, optic cup, early telencephalon and diencephalon P3 region.

The suggestion that the two copies of zebrafish *pax6* arose through whole genome duplication [72] led us to explore the current synteny relationship of each co-orthologue, with the expectation that we would find two genomic copies of the known closely linked human markers, *WT1*, *RCN1* and *ELP4* (Figure 4A). However, only one copy has been identified for each of these three closely linked genes in zebrafish at one or other of the expected syntenic sites [73] on either LG7 or LG25 (Figure 4A). Genomic localisation was confirmed for all three genes by mapping on the T51 radiation hybrid map [41,42], using specific primers designed from available zebrafish sequence data in public genome annotations. Assignments were confirmed on multiple BAC clones we had isolated for each *pax6* orthologue, including DKEYP-46C10 and DKEY-157G7 containing *pax6a* and *pax6b* respectively (Figures S4 and S5). These BACs were part of the zebrafish genomic sequence assembly, and are integrated into regional maps for LG7 and LG25, verifying the syntenic relationships on these linkage groups.

Zebrafish *wt1a* was linked to *z14229* as the nearest marker, upstream of *pax6a* on LG25 (Figures 4A and S4), showing well-conserved exon structure and sequence, in agreement with recently reported data [74]. A related *wt1* sequence was also found on LG18, close to marker *zc50f22*, but with no neighbouring *pax6* sequence (data not shown), although this region of LG18 is known to harbour a human chromosome 11 synteny region [73]. Sequences corresponding to the coding exons of *rcn1* and *elp4* were only found flanking *pax6b* on LG7 (Figures 4A and S5). This pattern of partial synteny conservation is best illustrated with a PIP plot [75] using the compact *Fugu* sequence as the baseline (Figure S6), which reveals the exonic sequence conservations very clearly: showing that *wt1a* is linked to *pax6a;* and *rcn1* and *elp4* to *pax6b* [67]. A similar PIP plot with the chick sequence as the baseline (Figure S7) shows unequivocally that the conserved elements associated with the downstream region of human and mouse *PAX6*, mapping within a number of the *ELP4* introns, are still present downstream of zebrafish *pax6a*, despite the loss of *elp4* exonic conservation. In contrast, most downstream elements have been lost from the pax6b locus where *elp4* has been maintained. This suggests that the whole syntenic region was ancestrally duplicated, but, in the absence of strong selective constraints, the *elp4* duplicate copy associated with *pax6a* was lost, presumably through multiple mutations (non-functionalisation), while the proposed *pax6* regulatory elements, formerly residing in its introns, have been selectively maintained. These finding clearly imply that these conserved elements are not involved in *elp4* RNA processing, a possible role suggested for intronic conserved sequences [76].

### Evolutionary Divergence of Regulatory Elements Associated with the Co-orthologues—Bioinformatic and Transgenic Reporter Analysis in Mouse and Zebrafish

Focusing on the evolving role of the duplicated zebrafish *pax6* genes, we observed multiple examples of subfunctionalization, or job-sharing, between *pax6a* and *pax6b*. The differential expression patterns of these co-orthologues are reflected by loss and retention of relevant conserved regulatory elements (Figure 4B).

**Figure 5:**
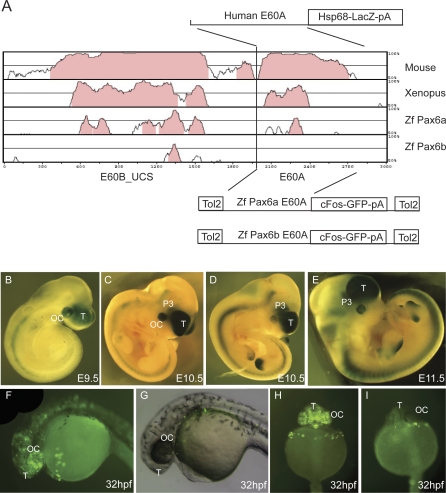
Enhancer Capacity of the E60A Conserved Element Assessed in Transgenic Mice and Zebrafish. (A) Vista plot of the region around the highly conserved region E60+, located 47 kb downstream from the PAX6 P1 promoter. Human sequence is used as the base for pairwise comparison with *pax6* loci from mouse, Xenopus tropicalis, and zebrafish (*pax6a* and *pax6b*). Fragment E60B_UCS contains an ultraconserved element that will be described elsewhere and is used here as an anchor point to allow localisation of the conserved/non-conserved E60A element from zebrafish *pax6a* and *pax6b*. A fragment containing the human E60A element was cloned into an hsp68-LacZ reporter construct to generate transient transgenic embryos. The equivalent regions from the zebrafish *pax6a* and *pax6b* loci were cloned into a Tol2 transposon based vector containing a cFos minimal promoter GFP reporter cassette [53] and used to produce transgenic zebrafish. (B–E) Reporter expression of the human E60A construct was observed in optic cup, telencephalon, and prosomere P3 region of the diencephalon in embryos of E9.5 (B); E10.5 (C and D); and E11.5 (E). Expression (ectopic) in the limbs is ascribed to the genomic integration site of the construct. (F) A similar GFP reporter expression pattern is seen in transgenic zebrafish embryos with the E60A region from the *pax6a* locus, with GFP expression observed in the optic cup and forebrain. (G) No reporter expression is seen with the construct containing the E60A region from locus *pax6b*. (H) Frontal view of a transgenic zebrafish with the *pax6a* E60A element, showing expression in telencephalon and optic cup. (I) Frontal view of a *pax6b* E60A transgenic fish showing a lack of specific expression. T, telencephalon; OC, optic cup; P3, prosomere P3 of the diencephalon.

In the PAX6 downstream region, in the large intron 9 of ELP4, we identified a new regulatory element, E60, showing 94% nucleotide sequence identity between human and mouse over 1400 bp, through sequence comparisons by PIP plot (Figures 4B and  S7). Two distinct subcomponents, E60A and E60B, can be distinguished, as shown (Figures 4B and 5A). E60B contains one of the largest ultra-conserved elements: 601bp of complete human-mouse identity [77]. Detailed VISTA analysis [78] revealed clear conservation of E60B in Xenopus and to some extent in both zebrafish *pax6a* and *pax6b* (Figure 5A). For E60A only pax6a showed discernible conservation (Figures 5A and S8A), while pax6b (Figure S8B) has no homologous conserved sequence. There have been recent suggestions that enhancer activity may be maintained even where no sequence conservation can be observed between mammals and teleosts [79]. We therefore set out to assess the function of the E60A region in parallel studies in both mice and zebrafish. A set of transient and permanent mouse transgenic reporter lines was produced, with the human E60A region (Figure S8A) as the enhancer, using previously described methods [6,7,9] (Figure 5B–5E). The transgenic mice show lacZ expression consistently in the early retina, in the telencephalon, and in the future ventral thalamic region of the diencephalon (Figure 5B), with minor ectopic expression seen only occasionally, for example, in the limb (Figure 5D and 5E). It is not clear whether the neural tube staining is due to the Hsp70 minimal promoter used.

The zebrafish E60A region (Figure S8A and S8B) from *pax6a* or *pax6b* (located using the conservation at the linked E60B element as an anchor point) was inserted, using Gateway cloning, into a Tol2 transposon based vector containing a cFos minimal promoter-GFP reporter cassette (Figure 5A) for co-injection with Tol2 mRNA, as described [53] (Figure S8C). The E60A region from *pax6a* gives rise to transgenic fish expressing GFP in the optic vesicle and the telencephalon (24/38 surviving transgenic embryos showed this expression pattern at 32 hpf in a typical experiment) (Figure 5F and 5H). No consistent or significant expression was seen in any of 23 surviving transgenic embryos similarly injected with the homologous *pax6b* construct (Figure 5G and 5I). In this instance, therefore, maintenance or loss of sequence conservation correlates strongly with the presence or absence of tissue-specific enhancer function across multiple species.

**Figure 6:**
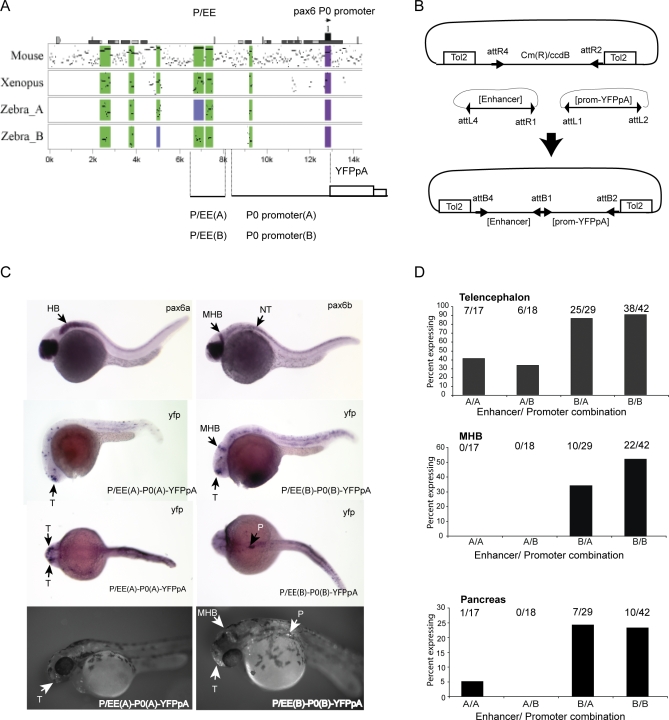
Transgenic Analysis of the Zebrafish P/EE Enhancers from the *pax6a* and *pax6b* Loci in Combination with the pax6 P0 Promoters (A) PIP plot showing the conservation levels in the *Pax6* upstream regions of mouse, Xenopus tropicalis, and the two zebrafish co-orthologues using the human gene as base sequence. The position of the pancreas/ectodermal (P/EE) and the P0 promoter regions used in the construction of the reporter transgenes are shown. (B) Schematic representation of the Tol2-2way system to create combinatorial constructs for Tol2 mediated zebrafish transgenesis. (C) Expression analysis of *pax6a* and *pax6b* shown by RNA in situ analysis with alkaline phosphatase staining at 28 hpf (top). YFP expression in P/EE(A)-P0(A)-YFPpA (left) and P/EE(B)-P0(B)-YFPpA (right) transgenic fish at 28 hpf shown, using in situ analysis for YFP mRNA. 56 hpf fluorescence images of YFP expression in transgenic lines for the same two constructs are shown on the bottom row. (D) Frequency of expression in different sites in transient transgenic zebrafish with the four combinations of P/EE enhancer and P0 promoter from the two *pax6* loci. T, telencephalon; MHB, midbrain hindbrain boundary; HB, hindbrain; P, pancreas; NT, neural tube.

Next, using zebrafish reporter transgenics, we assessed the roles of the compound element, P/EE, situated upstream of the two *Pax6* promoters, P1 and P0 in the mouse, and previously shown to include an ectodermal enhancer (EE) involved in regulation of Pax6 expression in lens, cornea and lachrymal gland development, and a pancreatic enhancer P (Figures 4B and S9) [36,65,80,81]. Conditional knockouts had confirmed the role of P/EE in both lens and pancreas development [36,80,81]. PIP plots [75] illustrate the conservation of the 5′ pancreas component [65] in the upstream region of *pax6b* and its absence in *pax6a* (Figures 2, 4B, and S7)*,* which is not expressed in pancreas (Figure S3) [43,49]. In contrast EE and an additional neighbouring element appear to be conserved in association with both *pax6a* and *pax6b*. The P/EE element from *pax6a* and *pax6b* failed to elicit GFP expression when either was inserted into the same c-fos minimal promoter containing vector as the two zebrafish E60A elements (Figure 5A). We therefore engineered a new Tol2 based vector system containing a two-way Gateway destination cassette which can accommodate a reporter cassette linked to the endogenous promoters in conjunction with a second fragment containing a putative *cis*-element (Figure 6B). Four different combinations of the zebrafish elements were created using the compound enhancer region and the P0 promoter from both *pax6a* and *pax6b* (Figure 6A and 6B). A similar size random sequence DNA was also linked to the zebrafish P0 region as control. YFP expression was assessed by fluorescence and also using RNA *in situ* analysis. The patterns of expression observed for the P/EE(A)-P0(A) (AA) and the P/EE(B)-P0(B) (BB) constructs are illustrated in Figure 6C. At 28 hpf the alkaline phosphatase-labelled YFP *in situ* results reveal expression patterns that recapitulate part of the endogenous *pax6a* and *pax6b* expression patterns in accordance with their divergent expression in pancreas and MHB. Frequencies of observed expression sites for the four combinations are shown in Figure 6D. All constructs show a high frequency of expression in the telencephalon, including the constructs with a random sequence linked to the P0 promoters, suggesting that telencephalon enhancer activity resides in the P0 promoter regions from both loci. Interestingly, the telencephalic expression frequency appears lower in the presence of P/EE from the pax6a locus. In contrast, expression at the MHB and at lower frequency in the pancreas are clearly dependent on the presence of P/EE(B). Constructs with the P/EE(A) element or a random sequence in combination with either promoter show reporter expression only in the telencephalon. Occasionally expression was found in a few isolated cells in the retina with the P/EE containing constructs. More detailed analysis of these expression patterns will be possible in transgene transmitting G1 and subsequent generations, when any transgene mosaicism will have been eliminated.

### Concluding Remarks

Gene duplication presents multiple discrete opportunities for differential alteration of gene function in closely related individuals. Divergence at just a few essential loci may lead to reproductive isolation and speciation, as in teleost fish [19–23]. Zebrafish *pax6a* and *pax6b* probably arose through large scale genome duplication, rather than regional duplication and translocation. Their position on different linkage groups, both showing vestiges of conserved synteny with ancestral neighbouring genes, is good evidence for this. Surprisingly, the *pax6* genes, which are strongly dosage sensitive in mammals [30,82–84], have survived duplication, while the ubiquitously expressed flanking genes *rcn1* and *elp4*, which do not give rise to a dosage (haploinsufficiency) phenotype in humans [85] or mice [68], have lost their co-orthologues. It is interesting to speculate whether the coordinated loss of tissue-specific regulatory elements in duplicate pax6 loci is selected for because gene dosage needs to be maintained at the correct level. Further studies in other teleost species may help to resolve this question.

The diverged functions of the *pax6* co-orthologues are unlikely to be due to coding region changes [43], although there are ∼20 amino acid differences between them, and also between each zebrafish gene and their single human orthologue. In contrast, the L244P mutation found in the *sri* mutant is deleterious; it shows reduced binding *in vitro* to a canonical paired-type homeodomain target site and its ability to activate both the canonical homeodomain target P3 and the paired domain target CD19 is abolished or severely reduced. In keeping with the expression spectrum of *pax6b*, the mutation gives rise predominantly to a microphthalmic phenotype with variable reduction in lens size and, in extreme cases, disorganisation of the retina (Figure 1A), that may be secondary to extreme lens reduction (cf Ashery-Padan et al, 2000 [80]). The absence of a severe pancreas phenotype is intriguing; our discussions raise various possible explanations, but further analysis is needed for better insight.

Our findings provide strong experimental evidence for the concept of evolutionary divergence by subfunctionalization through differential loss and retention of *cis*-regulatory elements at ancestrally duplicated tissue-specific loci [86]. This has also been termed the “duplication, divergence and complementation” model [87]. For the retention of a full set of functions, the loss of elements needs to proceed in an orderly manner. Interdependent *cis*-elements can only be lost from the same copy of the duplicated gene, otherwise gene activity in certain tissues may be totally lost. This may be the situation where several brain-specific elements, with spatiotemporal overlap in expression pattern, have been lost from *pax6b*. The persistence of *pax6a*-associated regulatory elements, when the exons of the *elp4* gene have disappeared, may be one mechanism for the evolution of gene deserts containing only conserved non-coding sequences [12,88].

## Methods

### Embryo collection.

General maintenance, collection, and staging of zebrafish was carried out according to the Zebrafish Book [89]. The approximate stages are given in hours post fertilization (hpf) at 28 °C and are determined according to morphological criteria [90].

### Genetic mapping of the sri locus.

The zebrafish mutant *sunrise* (allele: *sri^tq253a^*)*,* produced as part of an ENU mutagenesis screen [27], was provided by the Tübingen zebrafish stockcentre. Homozygous *sri* fish, showing the variable recessive phenotype, were found to be fully viable and fertile. Fish carrying the *sri* mutation on the Tübingen background were crossed to the wildtype strain WIK (provided by Peter Currie). The F1 was raised, and 5 dpf F2 larvae showing the mutant phenotype, and their WT siblings, were collected and fixed in 100% methanol prior to mapping. The *sri* mutation was mapped by linkage analysis with SSLP (simple sequence length polymorphism) markers between embryos showing the homozygous mutant phenotype and their unaffected siblings.

### Radiation hybrid mapping.

Radiation hybrid (RH) mapping was carried out using the Goodfellow T51 RH panel. To obtain the positions of the tested markers the Children's Hospital Zebrafish Genome Initiative webpage (http://zfrhmaps.tch.harvard.edu/ZonRHmapper/instantMapping.htm) was used. Intronic primers flanking exons 1 and 6 of *pax6b* were used to map this strong candidate gene onto the T51 RH map that was also used as a reference for the markers used in the genetic mapping. The map position of *pax6b* was closest to the marker *fc33g08*, which is now known to be part of the *pax6b* coding region in NM_131641 at http://www.ncbi.nlm.nih.gov/entrez/viewer.fcgi?db=nucleotide&val=18859210 .

### Mutation analysis using RT-PCR and sequencing.

*pax6b* cDNA was derived by RT-PCR from *sri* and WT embryos using Superscript II (Gibco). Sequencing revealed four changes identified in the *sri* coding sequence compared to the published *pax6b* cDNA [43] (Genbank NM_131641). Three of the four nucleotide changes were synonymous (429 A>C, 989 G>A, 1304 A>C), and also found to be present in the wildtype sequence of the Tü strain, in which the *sri* mutation was generated [15], as well as in the public genomic sequence in BAC DKEY-157G7. The fourth, a T to C transition at position 991 in the cDNA is present only in sri DNA and gives rise to a leucine to proline missense mutation in the homeodomain. The 991 T>C mutation creates a *DdeI* digestion site that was used for further genotyping (Figure S2).

### Functional studies of the sri mutant protein.

Luciferase assays were carried out as follows. Mammalian expression vectors, pCMV-ZfPax6.1 (pax6a), pCMV-ZfPax6.2 (pax6b) and pCMV-ZfPax6.2_*sri* (pax6b_sri), respectively containing the two wild type or L244P mutant full-length cDNA clone, were used in co-transfections into HeLa cells with the luciferase reporter plasmids 3XP3-Luc and 3XCD19-Luc, containing respectively three P3 homeodomain binding sites [45] or three CD19 paired domain binding sites [46] linked 5′ to the basal promoter-luciferase cassette. HeLa cells were cultured in DMEM medium with 10% calf serum (Gibco, Milan-Italy) and transfected using calcium phosphate co-precipitation [91]. Cells were plated at 6 × 10^5^ cells/100 mm culture dish 20h prior to transfection. The plasmids used were: pCMV-ZfPax6b and pCMV-Zf Pax6b*sri*: 1 μg; 3XP3-Luc: 9 μg; RSV-CAT: 2 μg. RSV-CAT contains the RSV promoter 5′ to the choramphenicol-acetyl-transferase (CAT) gene, used to normalize transfection efficiency. Cells were harvested 42–44 h after transfection, and extracts prepared by freeze-thawing. CAT activity was measured by ELISA (Amersham), Luciferase activity by chemiluminescence. The data presented are mean values plus/minus standard deviations of a representative experiment performed in triplicate (three independent transfections).

Electrophoretic mobility shift assays (EMSA) were conducted as follows. cDNA sequences encoding either wild-type ZF pax6b or mutant L244P homeodomain (from aminoacid 228 to amino acid 291 of the full-length protein) were cloned in the pT7.7 bacterial expression vector, checked by nucleotide sequencing, expressed in BL21 cells, and partially purified. ^32^P end-labeled double-stranded oligodeoxynucleotides P2 and P3 containing high-affinity HD-binding sequences

P2: 5′-TCGAGGGCATCAGGATGCTAATTGATTAGCATCCGATCGG -3′;

P3: 5′- TCGAGGGCATCAGGATGCTAATTGGATTAGCATCCGATCGG -3′

were used in gel-retardation assays [47,92]. HD-containing protein at a concentration of 50, 100 and 300 ng/sample was incubated for 30 minutes at room temperature with probe DNA (5 mM in a buffer containing 20 mM Tris-HCl pH 7.6 , 75 mM KCl, 0.25 mg/ml bovine serum albumin (BSA), 5mM dithiothreitol (DTT), 12.5 μg/ml calf thymus DNA, 10% glycerol). Protein-bound and free oligos were separated on native 7.5 % polyacrylamide gel, run in 0.5 × TBE for 1.5 hours at 4 °C. Gels were fixed and autoradiographed. Electrophoretic mobility shift assays were carried out for wild type and mutant homeodomain protein fragments on the P2 and P3 homeodomain binding sites.

For histological analyses, *sri* mutant and age-matched sibling larvae were fixed in 4% paraformaldehyde in PBS, dehydrated in a standard ethanol series, embedded in Technovit (Heraeus Kulzer, Germany), sectioned and stained with 0.5% toluidine blue in 1% borax buffer.

Immunohistochemistry was performed using standard methods and suitably cross-reacting antibodies. Mouse monoclonal anti-porcine glucagon antibody (Sigma G2654) and guinea-pig polyclonal anti-porcine insulin antibody (DAKO A0564) were used with fluorescently labelled second antibodies. Individuals studied were genotyped, as described.

Comparative sequence analysis was carried by through PIP plots, which were made using the programme PIPmaker (http://bio.cse.psu.edu/pipmaker/) [75]. Evolutionary sequence comparison of element E60+ was prepared using the VISTA program [78] with a window size of 50 bp and a minimal sequence identity of 70% using sequences derived from Z83001 (human), AL512589 (mouse), AL021531 (pufferfish), and AL929172, BX004784 (zebrafish pax6a), AC127461, BX000453 (zebrafish pax6b). Xenopus sequence was obtained from Xenopus tropicalis genome assembly 3.0 from the JGI.

### Expression studies of pax6a, pax6b, and YFP by RNA in situ analysis in zebrafish.

Preliminary information is in the public domain [49] and can be accessed directly at the ZFIN database (http://zfin.org/cgi-bin/webdriver?MIval=aa-xpatselect.apg) (Accessed: 16 January 2008).

Whole-mount *in situ* hybridization reactions were carried out according to published protocols [93]. The cDNA constructs were prepared through cloning of sequence verified RT-PCR products using RNA prepared from wild type and *sunrise* mutant zebrafish into the eukaryotic expression vector pJG1 (gift from Jacqueline Guy). Riboprobes for *pax6a* and *pax6b* were transcribed from PCR templates amplified from plasmid DNA using the following oligonucleotides:

pax6afw:5′-CCCAATACTGGCCCAGACTA-3′

pax6aT7rv:5′-TAAGCTTTAATACGACTCACTATAGGGAGAGAAGTGGCACTATCCCCGTA-3′

pax6bfw:5′-GAGCAAGATTCTGGGGAGGT-3′

^pax6bT7rv:5′-TAAGCTTTAATACGACTCACTATAGGGAGAGCTCGGTATGTTATCGTTGG-3′^

^eYFPfw: 5′-CACATGAAGCAGCACGACTT-3′^

^eYFPT7rv: 5′-TAAGCTTTAATACGACTCACTATAGGGAGAAGTTCACCTTGATGCCGTTC-3′^

Sections of whole-mount *in situ* stained embryos were obtained by wax embedding and sectioning at 7 μm .

### Expression analysis of adult zebrafish tissues by RT-PCR.

RNA was made from carefully dissected adult (age >6 months) pancreas from both wildtype and homozygous mutant sunrise fish with Trizol (Sigma), and reverse transcribed with AMV reverse transcriptase (Roche) and random hexamer primers. Wild type eye tissue was processed alongside as control tissue expressing both pax6a and pax6b. The resulting cDNA was PCR amplified with primer pairs specific for pax6a, pax6b or rcn1, a ubiquitously expressed calcium binding protein. Primers used: *pax6a* (5′-cagtacaagagggagtgtcc-3′ and 5′-ctcaccgcctccgtctgactgt-3′),

*pax6b* (5′-CAGTACAAGAGGGAGTGTCC-3′ and 5′-GTTTTCACCACCGTTGTCCTGT-3′)

and *rcn1* (5′-TTCCACGAGATGAACGCAGGT-3′ and 5′-TTCATCTTCATCGACATGACCATC-3′).

### Antisense morpholino oligonucleotide injections.

The *pax6b* antisense morpholino oligonucleotides (MO) (Gene Tools, LLC) were directed against the 5′ sequence near the start of translation [94]. The MO used were:

*pax6a* v1-MO:5-CGTTTACAATAACGAGAAGACTCTG-3 *pax6a* v2-MO:5-AGTTCCAACAGCCTTTGTATCCTCG-3 *pax6b* v1-MO:5-GCCTGAGCCCTTCCGAGCAAATCAG-3 *pax6b* v2-MO: 5-TGGTATTCTTTTTGAGGCATTCTGC-3

They were injected in a volume of 1.4 nl through the chorion of 1 to 2-cell stage embryos to deliver a mass of 3.5 ng. For each MO, at least 100 embryos were injected and the phenotype was of consistent severity and fully penetrant. Co-injection of pax6a and pax6b morpholinos was also tried.

### RNA injections for phenotype rescue.

The wild-type and *sri* mutant pax6b injection construct was cloned into pJG1 from fish-derived cDNAs as described above. The capped mRNA was synthetized using the mMessage mMachine kit T7 (Ambion) and purified using the RNeasy Mini kit (Qiagen). The RNAs were injected into 1 to 2-cell stage embryos as described previously.

### Reporter transgenesis in zebrafish using the Tol2 system.

The E60A_cfosEGFP and E60B_cfosEGFP (Figures 6 and S7C) were generated according to published protocols [53], using the following oligonucleotides:

*pax6a* E60afw: 5′-GGGGACAAGTTTGTACAAAAAAGCAGGCTAACAAATGTCACAGACTGCAAGA-3′

*pax6a* E60arv: 5′-GGGGACCACTTTGTACAAGAAAGCTGGGTGCGCGCCTACAACCACGTTTCTACCAGC-3′

*pax6b* E60afw: 5′-GGGGACAAGTTTGTACAAAAAAGCAGGCTACTGGAAAATATACAGAGGGC-3′

*pax6b* E60arv::5′-GGGGACCACTTTGTACAAGAAAGCTGGGTGCGCGCCATTCATCCGTCTGTCCATCCA-3′Fish were injected as described for RNA injections.

### Tol2-2way system to create combinatorial constructs for Tol2 mediated zebrafish transgenesis.

A vector was created in which an AttR4-R2 Gateway 2-way recombination cassette (Invitrogen) was flanked by *cis*-sequences required for Tol2 mediated transposition based on the medaka Tol2 transposon [53] generating the pTol2-2way destination vector. Putative enhancer regions are cloned into an attP4-P1r plasmid and combined with a promoter-reporter cassette containing attL1-L2 plasmid and the pTol2-2way vector to generate the microinjection construct. Various combinations of those two constructs can be recombined into the pTol2-2way destination vector The entry clones for the P/EE elements or the random sequence (RS; a non-conserved region in a gene desert on human chromosome 17) were made using the following primers:

attB4-P/EE(a) primer: 5′-AGGGGACAACTTTGTATAGAAAAGTTGGCGCGCCTCTCCGCCAATGAATCTGCA-3′

attB1r-P/EE(a) primer: 5′-AGGGGACTGCTTTTTTGTACAAACTTGTAGTTGGATATTACAGGCAGT-3′

attB4-P/EE(b) primer: 5′-AGGGGACAACTTTGTATAGAAAAGTTGGCGCGCCATGAACAGACAGATATGGCA-3′

attB1r-P/EE(b) primer: 5′- AGGGGACTGCTTTTTTGTACAAACTTGAACGTCCTGACCATGCAGGCT-3′

attB4-RS primer: 5′-AGGGGACAACTTTGTATAGAAAAGTTGGCGCGCCAGTCATTAGATCAATCCCT-3′

attB1r-RS primer: 5′-AGGGGACTGCTTTTTTGTACAAACTTGATTCTTGCTGGGTAGGGTACA-3′

The promoter-reporter constructs were made by cloning of the P0 promoter regions from the zebrafish pax6a (2 kb) and pax6b (2.5 kb) loci in front of a promoterless YFPpolyA cassette in an attL1-L2 containing pDONR vector, using an NcoI restriction site and the following primer sets:

*pax6a* P0 promoter: 5′-AACCATGGACTGCCTGTAATATCCAACTAC-3′ and 5′-ACCATGGTGGCGGCAGTCCAACAAGGGAACCTCGA-3′.

*pax6b* P0 promoter: 5′- ACCATGGATCTGCGACATGCTCATGTGA-3′ and 5′-ACCATGGCGGCCGGCCTGCTGTTTCAAGCCCTT-3′.

### Analysis of E60 reporter constructs in transgenic mice.

The E60A-Z reporter constructs are based on a modified p610 vector containing a hsp68-*LacZ* cassette [6]. A 2.3 kb fragment containing the E60+ conserved region was PCR amplified from human cosmid G0453 (EMBL Z83001) using high fidelity polymerase (Bio-X-Act) with primer pair (5′-TGTCGACCAATGCAGCACCAAAGTGTATGC-3′) (5′-AGTCGACAAAAGATAAGTAAGCTCAGATGT-3′), and cloned into the SalI site of the p610+ vector to generate E60+Z. The E60A-Z microinjection fragment was generated from this construct using the Asp718I restriction site located between the two subregions of the full-length E60+ fragment. All the expressions patterns were photographed on a Leica MZ FLIII Microscope fitted with a Hamamatsu Orca-ER digital camera.

## Supporting Information

Figure S1 Identification and Validation of the *sri* Mutation (A) Mapping of the *sri* mutation to the pax6b region on LG7. Markers used in the region are shown for a Radiation Hybrid (RH) map and by linkage.
(B) Alignment of the amino acid sequences for the PAX6 homeodomain with Pax6 proteins from multiple other species and with other human homeodomains from multiple genes with paired-type homeoboxes. The alignment shows (arrowed) that the L244P change has arisen in a totally conserved leucine residue.
(C) Documentation of the nucleotide differences (red boxes) between deposited genomic and cDNA sequences for *pax6b* and showing the position of the proposed ENU-induced CTT to CCT nucleotide change leading to the missense mutation. All the other nucleotide variants (strain differences) do not lead to protein residue changes.
(323 KB PPT)

Figure S2 Genotyping the Offspring of Two Heterozygous Parents by Restriction Analysis Reveals the Expected Numbers of Each Genotype (197 KB PPT)

Figure S3 RT-PCR Analysis Shows That Only *pax6b* Is Expressed in the Pancreas of Both Wild Type and sri/sri Homozygous Adult Fish. In Contrast, Both *pax6a* and *pax6b* Are Expressed in Adult Eye (199 KB PPT)

Figure S4 BAC Assembly of the LG25 *pax6a* Region, Showing the Synteny Conservation with *wt1a*
The other two immediate pax6 flanking genes normally seen in vertebrates, *elp4* and *rcn1*, are however absent from this contig.
(496 KB PPT)

Figure S5 BAC Assembly of the LG7 *pax6b* Region, Showing the Presence of *elp4* and *rcn1*
(56 KB PPT)

Figure S6 Sequence Comparison around *pax6* by PIP Plot, with *Fugu* as the Base Sequence, Showing That *elp4* Exons Are Present Downstream of Zebrafish pax6b, but Absent from *pax6a*
(199 KB PPT)

Figure S7 Sequence Comparison around *pax6* by PIP Plot, with Chick as the Base Sequence Comparing to Human, Mouse, *Fugu* and *pax6a* and *pax6b* from Zebrafish The enhancer elements discussed in this paper are highlighted with red boxes (P/EE, E60, and CE2). A blue box draws attention to the greater conservation of regulatory elements downstream of *pax6a* than *pax6b*, which is noteworthy since *pax6a* has lost the *elp4* exons, while pax6b has retained them.
(315 KB PPT)

Figure S8 Details of the E60 Elements in Mouse and Zebrafish Transgenic Studies (A) Mouse human and zebrafish comparisons for the E60A region.
(B) E60A sequence from the *pax6b* region, with no observable conserved sequence to *pax6a* E60A or to other vertebrate homologues, was tested in the zebrafish transgenics.
(C) The making of Tol2 constructs for efficient zebrafish transgenesis.
(163 KB PPT)

Figure S9 Summary of Conserved Elements within and Flanking the *PAX6* Gene, Most with Proven Tissues-Specific, *Pax6* Compatible Enhancer Function in the Mouse. (63 KB PPT)

### Accession Numbers

The RefSeq (National Center for Biotechnology Information [NCBI]) (http://www.ncbi.nlm.nih.gov/RefSeq) accession numbers for the genes discussed in this paper are for human: *PAX6* (NM_000280, NP_001595); *ELP4* (NM_019040); *RCN1* (NM_002901); *WT1* (NM_024425); mouse: *Pax6* (NM_013627); *Elp4* (NM_023876); *Wt1* (NM_144783); Zebrafish: *pax6a* (NM_131304); *pax6b* (NM_131641); *elp4* (NM_001017638); *rcn1* (predicted) (XM_686046); *wt1a* (NM_131046); *wt1b* (NM_001039634).

Diseases, etc.: human aniridia (MIM 106210); mouse small eye (MGI:1856155); zebrafish sunrise (ZDB-GENE-070117–2413).

## Abbreviations

dpf: days post-fertilisation
E60: highly conserved enhancer 60 kb downstream of PAX6 promoter
ELP4/ Elp4/ elp4: human, mouse and zebrafish elongation protein 4, regulatory component of RNA polymerase 2 complex)
ENU: ethyl-nitroso-urea (alkylating mutagen)
hpf: hours post-fertilisation
LG: linkage group
*MHB*: mindbrain-hindbrain boundary
P/EE: pancreas/ectodermal enhancer element
*PAX6/Pax6/pax6,*: human, mouse and zebrafish paired domain and homeodomain protein 6 gene
*RCN1/ Rcn1/ rcn1* human: mouse and zebrafish reticulocalbin 1
*sri,*: sunrise small eyed phenotype in zebrafish
*WT1/ Wt1/ wt1* human: mouse and zebrafish Wilms tumour predisposition gene
